# Type Genomics: A Framework for Integrating Genomic Data into Biodiversity and Taxonomic Research

**DOI:** 10.1093/sysbio/syaf040

**Published:** 2025-05-20

**Authors:** Harald Letsch, Carola Greve, Anna K Hundsdoerfer, Iker Irisarri, Jenna M Moore, Marianne Espeland, Stefan Wanke, Umilaela Arifin, Mozes P K Blom, Carolina Corrales, Alexander Donath, Uwe Fritz, Gunther Köhler, Patrick Kück, Sarah Lemer, Ximo Mengual, Nancy Mercado Salas, Karen Meusemann, Anja Palandačić, Christian Printzen, Julia D Sigwart, Karina L Silva-Brandão, Marianna Simões, Madlen Stange, Alexander Suh, Nikolaus Szucsich, Ekin Tilic, Till Töpfer, Astrid Böhne, Axel Janke, Steffen U Pauls

**Affiliations:** Department of Botany and Biodiversity Research, University of Vienna, Rennweg 14, 1030 Vienna, Austria; Staatliches Museum für Naturkunde, Erbprinzenstr. 13, 76133 Karlsruhe, Germany; Senckenberg Research Institute and Natural History Museum Frankfurt/M, Senckenberganlage 25, 60325 Frankfurt am Main, Germany; Senckenberg Natural History Collections Dresden, Museum of Zoology, Königsbrücker Landstr. 159, 01109 Dresden, Germany; Departamento de Biodiversidad y Biología Evolutiva, Museo Nacional de Ciencias Naturales (MNCN-CSIC), José Gutiérrez Abascal 2, 28006 Madrid, Spain; Leibniz Institute for the Analysis of Biodiversity Change, Museum of Nature Hamburg, Martin-Luther-King-Platz 3, 20146 Hamburg, Germany; Leibniz Institute for the Analysis of Biodiversity Change, Museum of Nature Hamburg, Martin-Luther-King-Platz 3, 20146 Hamburg, Germany; Leibniz Institute for the Analysis of Biodiversity Change, Museum Koenig Bonn, Adenauerallee 127, 53113 Bonn, Germany; Senckenberg Research Institute and Natural History Museum Frankfurt/M, Senckenberganlage 25, 60325 Frankfurt am Main, Germany; Departamento de Botánica, Instituto de Biología, Universidad Nacional Autónoma de México, 3er Circuito de Ciudad Universitaria, Coyoacán, Mexico City 04510, Mexico; Institute of Ecology, Evolution & Diversity, Goethe-University Frankfurt, Max-von-Laue-Str. 13, 60438 Frankfurt, Germany; Leibniz Institute for the Analysis of Biodiversity Change, Museum of Nature Hamburg, Martin-Luther-King-Platz 3, 20146 Hamburg, Germany; Museum für Naturkunde, Leibniz Institute for Evolution and Biodiversity Research, Invalidenstr. 43, 10115 Berlin, Germany; Leibniz Institute for the Analysis of Biodiversity Change, Museum Koenig Bonn, Adenauerallee 127, 53113 Bonn, Germany; Leibniz Institute for the Analysis of Biodiversity Change, Museum Koenig Bonn, Adenauerallee 127, 53113 Bonn, Germany; Senckenberg Natural History Collections Dresden, Museum of Zoology, Königsbrücker Landstr. 159, 01109 Dresden, Germany; Senckenberg Research Institute and Natural History Museum Frankfurt/M, Senckenberganlage 25, 60325 Frankfurt am Main, Germany; Leibniz Institute for the Analysis of Biodiversity Change, Museum Koenig Bonn, Adenauerallee 127, 53113 Bonn, Germany; Leibniz Institute for the Analysis of Biodiversity Change, Museum of Nature Hamburg, Martin-Luther-King-Platz 3, 20146 Hamburg, Germany; Leibniz Institute for the Analysis of Biodiversity Change, Museum Koenig Bonn, Adenauerallee 127, 53113 Bonn, Germany; Leibniz Institute for the Analysis of Biodiversity Change, Museum of Nature Hamburg, Martin-Luther-King-Platz 3, 20146 Hamburg, Germany; Leibniz Institute for the Analysis of Biodiversity Change, Adenauerallee 127, 53113 Bonn, Germany; Natural History Museum Vienna, Burgring 7, 1010 Vienna, Austria; Department of Biology, Biotechnical Faculty, University of Ljubljana, Jamnikarjeva ulica 101, 1000 Ljubljana, Slovenia; Senckenberg Research Institute and Natural History Museum Frankfurt/M, Senckenberganlage 25, 60325 Frankfurt am Main, Germany; Senckenberg Research Institute and Natural History Museum Frankfurt/M, Senckenberganlage 25, 60325 Frankfurt am Main, Germany; Institute of Ecology, Evolution & Diversity, Goethe-University Frankfurt, Max-von-Laue-Str. 13, 60438 Frankfurt, Germany; Leibniz Institute for the Analysis of Biodiversity Change, Museum of Nature Hamburg, Martin-Luther-King-Platz 3, 20146 Hamburg, Germany; Senckenberg Research Institute and Natural History Museum Frankfurt/M, Senckenberganlage 25, 60325 Frankfurt am Main, Germany; Leibniz Institute for the Analysis of Biodiversity Change, Museum Koenig Bonn, Adenauerallee 127, 53113 Bonn, Germany; Leibniz Institute for the Analysis of Biodiversity Change, Museum Koenig Bonn, Adenauerallee 127, 53113 Bonn, Germany; Natural History Museum Vienna, Burgring 7, 1010 Vienna, Austria; Senckenberg Research Institute and Natural History Museum Frankfurt/M, Senckenberganlage 25, 60325 Frankfurt am Main, Germany; Leibniz Institute for the Analysis of Biodiversity Change, Museum Koenig Bonn, Adenauerallee 127, 53113 Bonn, Germany; Leibniz Institute for the Analysis of Biodiversity Change, Museum Koenig Bonn, Adenauerallee 127, 53113 Bonn, Germany; Institute of Ecology, Evolution & Diversity, Goethe-University Frankfurt, Max-von-Laue-Str. 13, 60438 Frankfurt, Germany; Senckenberg Biodiversity and Climate Research Centre, Senckenberganlage 25, 60325 Frankfurt am Main, Germany; Senckenberg Research Institute and Natural History Museum Frankfurt/M, Senckenberganlage 25, 60325 Frankfurt am Main, Germany; Institute of Insect Biotechnology, Justus-Liebig-University Gießen, Heinrich-Buff-Ring 26-32, 35392 Gießen, Germany

**Keywords:** Ancient DNA, biodiversity, DNA barcoding, historical DNA, holotype, natural history collections, taxonomy, whole-genome sequencing

## Abstract

Name-bearing type specimens have a fundamental role in characterizing biodiversity, as these objects represent the physical link between a scientific name and the biological organism. Type specimens are usually deposited in natural history collections, which provide key infrastructure for research on essential biological structures and processes, while preserving records of biodiversity for future generations. Modern systematics increasingly depends on genetic and genomic data to differentiate and characterize species. While the results of genome sequencing are often connected to a physical voucher specimen, they are rarely derived from the ultimate taxonomic reference for a species, that is, the name-bearing type specimens. This is a known but underappreciated problem for ensuring the replicability of findings, especially those that affect the interpretation of biodiversity distributions and phylogenetic relationships. Destructive sampling of museum specimens, particularly of type material, often carries a high risk of sequencing failure, and thus the cost of damage to the specimen may outweigh the resulting benefit. Both taxonomic work and genome sequencing require specialist skills, and there are often communication gaps between the respective experts. A new, harmonized approach, maximizing information extraction while minimizing risk to type specimens, is a critical step forward toward linking disciplines across biodiversity research and promoting a better taxonomic and systematic understanding of eukaryotic diversity. The genetic makeup of a type specimen is a fundamental part of its biological information, which can and should be made freely and digitally available through type genomics. Here, we describe guidelines for the use of nomenclatural types in genome sequencing approaches, considering different kinds of types in different stages of preservation and different data types.

All biodiversity knowledge is rooted in the concept of the species as the fundamental biological unit. Species are defined by name-bearing type specimens, which are usually, but not necessarily, typical of individuals belonging to that species (Haber 2012; Sluys 2021). The name-bearing type specimen thus represents the physical link to the scientific name (ICZN 1999; Thomson et al. 2018; Turland et al. 2018). It is the definitive reference point for species identity. Type specimens are usually deposited in natural history collections (NHCs) in museums, research, and biobank institutions. These collections form the basis for taxonomic and systematic research focusing on the identification, nomenclature, and classification of species, providing the basis for repeatable interpretation of phenotypes (Hilton et al. 2021). Beyond their role as repositories for (type) specimens, NHCs are important resources for other fields of basic and applied research, as they represent our archive of biodiversity across time and space. These include biodiversity dynamics (e.g., evolutionary biology, conservation, ecology), the origin and spread of invasive species (including pathogens), community responses to environmental change, and national and international monitoring programs (Yang et al. 2011). NHCs therefore support research on fundamental biological structures and processes (Beck 2018). The practice of depositing specimens in NHCs started over 200 years ago. Today, research museums and herbaria house several million primary type specimens (e.g., Franzen and Glaw 2007; Smith et al. 2013; Türkay et al. 2018; Bassini-Silva et al. 2021). The value of these types lies not in the morphological, genetic, or ecological information associated with it, but in the fact that it allows to unmistakably assign this information to a scientific name. NHC curators have a duty to balance two, often conflicting, obligations: preserving the physical integrity of the type specimens and ensuring that these specimens and the information they contain are accessible to the scientific community and society. In outlining this challenge, we need to keep in mind that its extent varies dramatically between different types of organisms, where tiny species with singular morphological features are the most prone to being destroyed through physical access, but may also be the species where physical access is so important.

Fulfilling conservatory duties is challenging due to the limitations of long-term preservation methods used in NHCs as well as financial constraints on collections infrastructure and staff. Even under ideal storage conditions, the physical integrity of type specimens degrades over time due to oxidation, light, and hydrolysis, which particularly affects DNA preservation (Miller et al. 2013). Furthermore, types can be completely destroyed by pests (insects, fungi, and bacteria), natural disasters, fire, or military conflicts (e.g., AAAS News 1978; TNN 2016; Lopes 2018; Tylor et al. 2023). The duty to safeguard the physical integrity of type specimens can be at odds with the curators’ second obligation to ensure their accessibility. Both the research use and transport of type specimens between institutions on loan carry a substantial risk of loss or damage. However, ensuring access to type specimens is essential to science, and an inaccessible NHC has no scientific value. Digital access to high-quality specimen images and metadata enhances accessibility and minimizes impact; however, this information is not sufficient on its own for many groups of organisms where microscale or internal morphological details are necessary for their characterization. Further expanding digital specimen information to include high-resolution digital 2D and 3D images, observational, morphometric, and bioacoustic information (Gemeinholzer et al. 2020), and genetic or genomic resources provides broad and equitable access to specimen data, and is essential to the long-term preservation of biodiversity information (“the extended specimen”; Webster 2017; Lendemer et al. 2020; Miralles et al. 2020; Hardisty et al. 2022; Davis et al. 2023). Comprehensive digital genetic data can be shared and analyzed without repeated sampling of the physical specimen. Genome-wide digital sequence information for type specimens provides additional informative characters for species identification that ultimately underlie phenotypic characteristics. Integrating these data into a “digital twin,” in the sense of a virtual representation of the physical type specimen (Kuhn 2017; Trauer et al. 2020), enables access to scientifically informative specimen data and interconnection of scientific and historical data (Sigwart et al. 2025), while ensuring the safe preservation of the original material, creating a digital reference in case of physical damage to the specimen, and increasing the research value of museum specimens.

Renner et al. (2024) presented a detailed account of the incorporation of sequences derived from type specimens in the National Center for Biotechnology Information (NCBI) GenBank. They elucidated the corresponding processes and difficulties involved in the comprehensive and reliable storage of type-derived sequences in databases. The article emphasizes the crucial role of “type sequences” as references in obtaining an accurate taxon identification via BLAST searches. Renner et al. (2024) demonstrated that GenBank currently contains a relatively limited number of sequences derived from eukaryotic type material. Furthermore, these sequences are typically short fragments of mitochondrial or intron-free nuclear genes. This is particularly evident for sequences originating from older museum material, from which the sequencing of DNA remains challenging. Renner et al. (2024) argue that sequencing eukaryotic type specimens is difficult due to the poor preservation of DNA in most historical specimens. Historically, this is undoubtedly correct, as generating Sanger or third-generation long-read sequences depends on high concentrations of long DNA fragments as input. With massive short-read sequencing technologies and the continuous development of methods to generate genomic resources from ever-smaller amounts of DNA, either from native DNA or following whole-genome amplification (e.g., Raxworthy et al. 2021; Schneider et al. 2021; Fong et al. 2023), a paradigm shift is now possible. We propose “type genomics”: a broad-scale, cross-taxon effort to sequence complete genomes or “low coverage” genomic data for type specimens. Here, we examine the technical and infrastructural opportunities and challenges of “type genomics,” and outline the potential benefits. The program we present here expands upon the plea by Renner et al. (2024) to increase publicly available type specimen-associated genomic information in light of emerging sequencing technologies.

## Extending the Specimen with Genomic Resources

While a complete and accurate record of global biodiversity requires comprehensive descriptions of the name-bearing specimens, incorporating all available evidence, a focus on taxonomically informative characters in the form of short diagnoses may be useful to cope with the exorbitant number of yet undescribed species. Taxonomy, the science of describing species and natural groups of species, has always depended on the integration of multiple lines of evidence to find reliable diagnostic characters that identify and separate species as distinct ancestor-descendant lineages (Sigwart 2018). The integration of molecular techniques has long been proposed for species identification and delimitation, with the goal of facilitating and accelerating taxonomy (Hebert et al. 2003; Tautz et al. 2003; Karbstein et al. 2024). Neither the International Code of Zoological Nomencl (ICZN) nor the International Code of Nomenclature for algae, fungi, and plants (ICN) requires molecular sequence data for the valid publication of new species. However, a growing number of authors use them as easily available and often unambiguous diagnostic features (see review by Renner 2016).

In cases of doubtful species assignment (e.g., cryptic species, polymorphism, phenotypic plasticity), sequence data can help resolve uncertainties arising from solely morphology-based taxonomy (e.g., Stuart and Fritz 2008; Strutzenberger et al. 2012; Petzold et al. 2014; Mutanen et al. 2015; Hausmann et al. 2017; Hundsdoerfer et al. 2017; Hartop et al. 2022; Islam et al. 2024). In animals, plants, and fungi, genetic identification is frequently achieved by providing the sequence of “barcode” gene fragments (Hebert et al. 2003; CBOL working group 2009; Schoch et al. 2012; Riedel et al. 2013; Hollingsworth et al. 2016). Today, type specimens of newly established taxa are often sequenced for these and other mitochondrial (e.g., Stuart and Fritz 2008; Funk et al. 2011; Hasan et al. 2012; Petzold et al. 2014; Köhler et al. 2021), plastid (Kress 2017), or nuclear (Irisarri et al. 2021) markers. In some cases, the complete organellar genome (e.g., Boo et al. 2016; Kehlmaier et al. 2019, 2020, 2023, 2024; Pätzold et al. 2021; Heckenhauer et al. 2023) or additional single nuclear loci (Hollingsworth et al. 2016; Kehlmaier et al. 2023) are provided to serve as a reference for phylogenetic and taxonomic studies. However, barcoding markers have serious limitations in taxonomic research that largely stem from their single-locus nature; for example, plastid- or mito-nuclear discordance or introgression (e.g., Funk and Omland 2003; Ballard and Whitlock 2004; Moritz and Cicero 2004; Currat et al. 2008; Toews and Brelsford 2012; Schniebs et al. 2016; Kehlmaier et al. 2019; Dietz et al. 2023; Wanke and Wicke 2023; Khan et al. 2024), Nuclear-mitochondrial DNA segments (NUMTs) (Bensasson et al. 2001), or heteroplasmy (Rubinoff et al. 2006). While DNA barcoding thus provides a valuable tool for species identification and taxonomy, its limitations highlight the need for integrative approaches that combine a genomic perspective together with morphological and ecological data to achieve the most comprehensive “extended type specimen.”

Incorporating genome-wide information in species descriptions overcomes the limitations and pitfalls of single locus sequences. Beyond anchoring the identity of a sequenced specimen or phylogenetic position of a species, genomic data can flexibly address a variety of evolutionary and taxonomic questions and inform conservation approaches such as macrogenetics, which utilizes already existing molecular data on a population level across taxa, space, and time (Leigh et al. 2021). The ever-decreasing costs of 2nd- and 3rd-generation sequencing and constant methodological improvements allow simple and cost-effective acquisition of hundreds or thousands of genetic loci, even from older or poorly preserved specimens (e.g., Staats et al. 2013; Derkarabetian et al. 2019; Mayer et al. 2021; Heckenhauer et al. 2023; Schöneberg et al. 2023). For example, a short-read, 10- to 20-fold coverage genome assembly, or a draft contig-level genome assembly, can (a) confirm species identity and phylogenetic placement with higher resolution, (b) inform on genome-wide patterns of divergence and presence of gene flow/conflict, (c) provide valuable population genetic/genomic information at the time of collection, and (d) encode additional fundamental information on the evolution and biology of the species (e.g., Köhler et al. 2021; Ronco et al. 2021; Twort et al. 2021; Deng et al. 2022; Razuri-Gonzales et al. 2024). Even draft genome assemblies such as these are sufficient to extract almost all protein-coding genes, including the benchmarking universal single-copy orthologs (BUSCOs), complete organellar genomes, and single nucleotide polymorphisms (SNPs) for phylogenomics and comparative genomics, as well as to infer key metrics of genetic variation such as heterozygosity.

**Figure 1.**
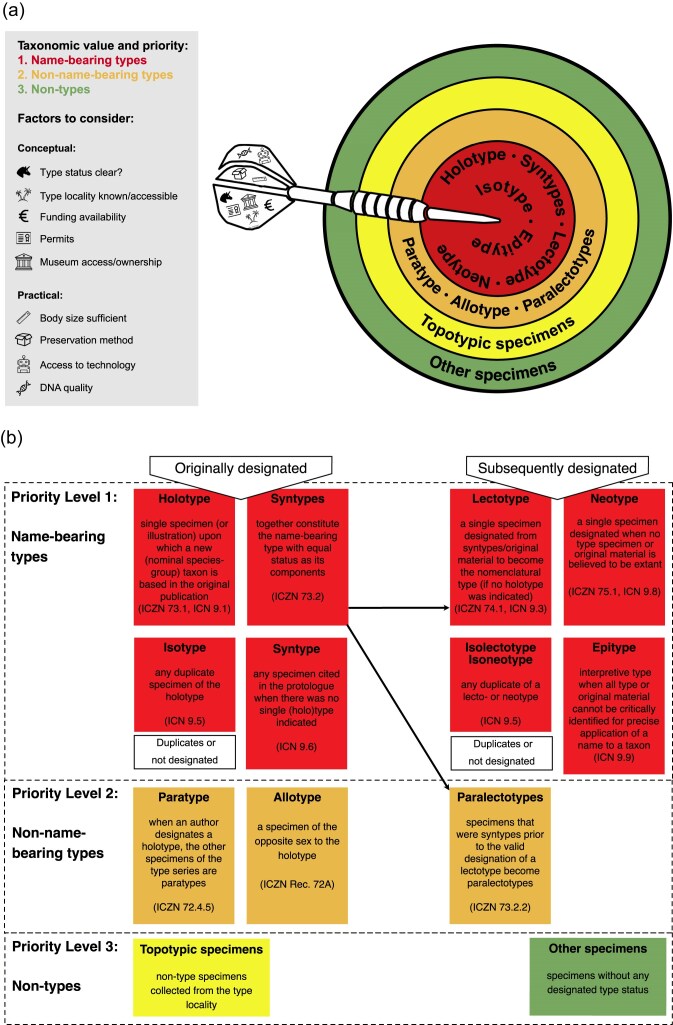
a) Target diagram reflecting the prioritization for genome sequencing according to taxonomic value (highest in the center; red). The priority level that can be reached depends on a number of considerations (shown in the dart and legend). The type series or original material (red and orange) might consist of i) a holotype and possibly para- or isotypes; ii) a lectotype and para- or isolectotypes; iii) syntypes; or iv) a neotype or epitype might have been designated. If no types are available, then a topotypic specimen (from the type locality) is preferred (yellow) over other specimens of the same species (green). After sequencing, further taxonomic treatments may be desirable, such as the designation of a sequenced lectotype from the syntype series or the designation of topotypic specimens as neotypes, if justified upon loss of the original types (see taxonomic implications). b) Flowchart representing the different type definitions. Black arrows indicate that lectotypes and paralectotypes are designated from syntype series.

Hence, whenever possible, genome sequencing, even with short-read technologies, should become a standard in the characterization of both new and existing name-bearing type specimens. Type genomics permanently preserves genome-scale information for types and enhances the utility and accessibility of the digital specimen (e.g., Mayer et al. 2021; Heckenhauer et al. 2023; Palandačić et al. 2023; Schöneberg et al. 2023; Razuri-Gonzales et al. 2024). This ensures that a genomic fingerprint of a type is permanently captured and preserved for posterity, even in the event of degradation or loss of said specimen. Several factors determine which specimens should be prioritized for whole-genome sequencing (WGS) (Fig. 1a). Notably, genomic data from specimens with very different taxonomically defined priorities can be very useful. For example, low-quality genomic resources derived from old or degraded name-bearing type specimens can phylogenetically anchor higher-quality genomes of non-name-bearing type or non-type specimens that, in turn, can be used in downstream systematic or comparative genomic analyses. Complete and manually curated mitochondrial and plastid genomes can also be assembled from “low coverage” sequencing data and used for phylogenomic and aid taxonomic decisions (Árnason et al. 2018).

A chromosome-level reference genome has become the ultimate information source for genomics to study species evolution, phylogenetic history, biogeography, taxonomy, genetics, population history, or inter- and intraspecific genetic diversity relevant for biodiversity conservation (Hoban et al. 2020). Comparative genomics also provides an opportunity to predict taxonomically relevant characters from genomic data and build on a growing understanding of the genotype–phenotype relationship (e.g., van’t Hof et al. 2011; Hiller et al. 2012; Nadeau et al. 2016; Van Belleghem et al. 2017; Weighill et al. 2019; Mazo-Vargas et al. 2022). As numerous initiatives to sequence genomes for all life on Earth progress (Lewin et al. 2022), ever more high-quality genomes are becoming available and open up the possibility to link newly sampled specimens to genomic resources (Böhne et al. 2024). Chromosome-level genomes are an important resource onto which short-read data from type specimens might be mapped, thus increasing the utility of these data. As researchers in taxonomy and genomics, our objective is not only to provide morphological characterizations of species but also to preserve genomic information for name-bearing types and advance the field of type genomics. Ideally, this information will ultimately be presented jointly in genomic databases, thereby providing a taxonomic and biological anchor for representative high-quality reference genomes.

## Maximizing Genomic Information from Types

In the following, we outline our proposed strategy for sequencing name-bearing type specimens. While this strategy cannot provide a universal solution for dealing with the wide variety of eukaryotic material and preservation methods, nor can it decide for the curator whether material is available for sequencing efforts, it can highlight important questions to be addressed prior to sequencing and, once a sequencing attempt has been made, in maximizing the potential output of genomic information.

### Evaluating Type Material Prior to DNA Extraction

**Figure 2.**
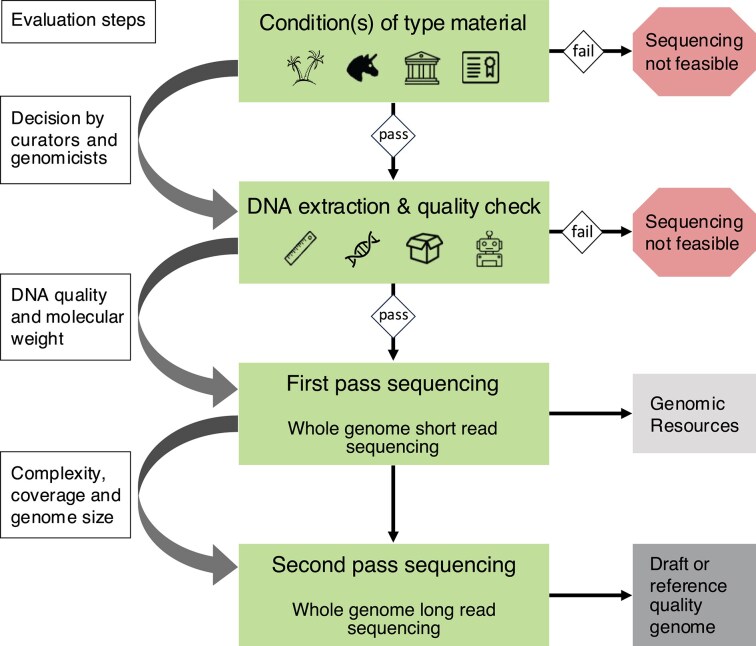
Flow chart representing the decision process on type sequencing. Icons correspond to legend in Figure 1.

Considering the unique value of name-bearing type specimens, potentially destructive sampling, even if minimally invasive, should only be considered if certain preconditions are met (Figs. 1 and 2). These include conceptual and practical considerations. Is the taxonomic status of the type specimen in question clear? Does the NHC or other repository have clear ownership and permits that allow for DNA extraction and sequencing of the specimen in question? Are the appropriate funds and facilities available to warrant a high likelihood of sequencing success? Only after positive evaluation of these questions should one proceed to extract DNA.

### DNA Extraction and Quality

Type specimens are unique, and careful treatment during the DNA extraction process is mandatory. The appropriate extraction strategy for type specimens depends on age, preservation and fixation methods, size, and storage conditions over time. It is therefore desirable to first proceed with methodological refinement using non-type material in a similar state of preservation to prevent irreversible damage to type material with a low probability of successful sequencing. One still has to keep in mind that even this approach does not guarantee ultimate success of DNA extraction and sequencing, as apparently similarly preserved material can give substantially different sequencing results (Straube et al. 2021a ; Bein et al. 2025).

Obviously, minimally invasive extraction methods should be standard when sampling museum specimens, especially types. To achieve this, non-destructive DNA extraction methods such as eraser samples of herbarium material (Shepherd et al. 2017), DNA extraction from (storage) liquid supernatant, or application of lysis buffers to specimens that leave material intact (Gilbert et al. 2007; Kirse et al. 2023) have been proposed. Some of these approaches have been successfully applied to historical specimens (Böhm et al. 2011; Hofreiter 2012; Giantsis et al. 2016; Pätzold et al. 2020; Timm et al. 2021; Cilia et al. 2022; Rayo et al. 2024). An alternative strategy to minimize specimen damage is to use leftover tissue from morphological or histological preparations for DNA extraction (e.g., genital preparations in insects; Hundsdoerfer and Kitching 2010; Pauls et al. 2010).

For smaller dried organisms, such as insects, minimal-invasive extraction protocols have demonstrated that it is possible to extract DNA from either the entire specimen (e.g., Santos et al. 2018) or one or more body parts without compromising the integrity of the specimen or its parts (e.g., Pauls et al. 2010; Rayo et al. 2024). However, multiple sampling attempts are likely to result in unacceptable levels of damage to the type. Further development and standardization of DNA extraction methods are necessary to increase success, reliability, and robustness, as is further testing with problematic samples, for example, tiny, formalin-fixed, or degraded tissues containing polysaccharides or other copurified substances, or subfossil bones. Supplementary Table S1 provides an overview of various protocols for ancient DNA (aDNA) and historical DNA (hDNA) extraction that have been used for DNA analyses of type specimens, differentiated according to tissue type and including references. Some protocols account for the degraded condition of the DNA and focus on extracting targeted or random small fragments, while others apply standard methods for preserving high molecular weight (HMW) DNA with valid results. The degree of degradation will differ for each sample according to its handling, fixation, and storage history (Fong et al. 2023).

In larger organisms, such as mammals, birds, and most plants and fungi, the amount of tissue may be less limiting (Särkinen et al. 2012). For these specimens, tissue samples can be taken from prepared dry skins or toe pads, or from leaf punches and stem tissue removal from herbarium sheets. In some cases, even multiple attempts are possible without substantial damage to the specimens, though tissue sampling from small organisms often requires expertise on which structures are taxonomically informative. For herbarium samples, destructive sampling should be used carefully to avoid damaging important diagnostic structures, and only the necessary amount of material should be collected (Davis et al. 2025). Furthermore, the designation of epitypes (e.g., for very small or unicellular types or types in the form of illustrations) may facilitate DNA extraction from living organisms such as material conserved in botanical gardens, but requires taxonomic and nomenclatural expertise and traceable label and origin information.

The quality of the extracted DNA also depends heavily on storage conditions, including duration, humidity, and temperature, factors that in many cases may be unknown for the past. Prior chemical treatment of specimens, such as formalin fixation of animal bodies or ethanol vapor to freshly collected plants, complicates DNA extraction. For example, in some groups, such as reptiles, amphibians, and fishes, more invasive sampling may be needed to extract tissue samples from internal organs with lower formalin penetration (e.g., Hahn et al. 2022). Nevertheless, an increasing number of studies show the possibility of successful DNA extraction (and analyses) despite cross-linking damage by formalin (Hoffman et al. 2015; Kehlmaier et al. 2020; Straube et al. 2021a). An example of the successful application for a historical lizard specimen was published by Kehlmaier et al. (2020) and for a fish type by Straube et al. (2021b). In addition, the chemical treatment of herbarium sheets, mammalian skins and bones, and insect collections to prevent pest attack can impede the extraction and amplification of DNA (e.g., Espeland et al. 2010). In ethanol-preserved material, age, storage conditions (e.g., ethanol purity), and organism size affect the amount and quality of DNA that can be extracted (Straube et al. 2021a). The worst-case scenario for type genomics is a collection of old, formalin-fixed small organisms stored in an ambient environment without climate control, which are typical conditions for most NHCs of soft-bodied invertebrates. Success may not yet be possible in such collections, and therefore exploratory projects as outlined in Figure 2 may be conducted, while broad-scale efforts may await further methodological development.

Curators should consult other taxonomic experts to proactively sample tissues from type specimens for cold storage and dehydration (Fig. 2) to prevent further degradation of DNA in historical specimens and preserve such samples for future technological advances. For example, the Museum of Comparative Zoology in Harvard has sampled and cryopreserved all types of the herpetological collection and 20% of invertebrates to minimize DNA degradation and as a backup of tissue in case of disaster (Card et al. 2021).

### First-Pass Sequencing (Short-Read, Low-Coverage Genomes or HybSeq)

Following DNA extraction, we recommend first-pass short-read sequencing as an initial attempt to generate a genomic resource. Recent high-throughput short-read sequencing (HTS) provides a cost-effective way to sequence organellar or nuclear genomes (Grayson et al. 2017) from type specimens. HTS has recently facilitated the pervasive use of reduced representation genome sequencing, such as target enrichment (TE) sequencing, as well as WGS. The former focus on sequencing only a subset of the genome rather than the entire genome to decrease the complexity and cost of sequencing by targeting specific regions of interest. The principle behind these techniques is to generate a sample that accurately reflects the genome by selectively capturing and sequencing specific regions. These methods have already been successfully applied to museum specimens (e.g., Staats et al. 2013; Derkarabetian et al. 2019)

TE methods use DNA probes to hybridize and selectively capture specific targets from a genome (e.g., Lemmon and Lemmon 2013; McCormack et al. 2012). This reduces complexity and the likelihood of sequencing contaminant DNA. Designing these probes is time-consuming and requires prior genomic knowledge of the taxa or close relatives. Furthermore, the compatibility of the targets with other data sets is not necessarily guaranteed. However, in the context of sequencing type material, the compatibility of the markers is mandatory. It is therefore recommended to rely on universal markers for which predesigned probe sets are already available, for example, ultra-conserved elements (UCEs; Bejerano et al. 2004; Faircloth et al. 2012), universal single-copy orthologs (USCOs; Eberle et al. 2020 ), or Hyb-Seq probe sets such as Angiosperm353 (Johnson et al. 2019).

Although the reduced-representation approaches have been shown to provide reliable markers, WGS based on short-read data can provide genomic characterization without the need to target specific markers a priori. It is therefore principally more versatile and maximizes the use of the acquired data. In terms of material preparation and laboratory protocol, WGS is also more convenient compared to TE (Allen et al. 2017; Call et al. 2024), as it does not require a target capture step. However, the advantage of TE over WGS is that the use of target-specific markers reduces the sequencing of contaminants. Both methods are suitable for small organisms (size < 1 mm, DNA < 10 ng) and for samples with low DNA concentration (e.g., Mayer et al. 2021; Call et al. 2024). High-coverage WGS is still relatively expensive, and costs rise with increasing genome size. In contrast, low-coverage WGS is cost-effective for organisms with small to medium-sized genomes and can then be applied to a large number of specimens. This makes it ideal for sequencing a larger amount of nuclear genes for many type specimens in NHC. Furthermore, organelle genomes are sequenced as a by-product, often with high coverage, since they are small and present in high copy numbers.

In plants, Hyb-Seq methods allow the simultaneous generation of nuclear, biparentally inherited loci through TE, along with uniparentally inherited organellar data via genome skimming (Weitemier et al. 2014). A combination of enriched and non-enriched libraries in a single pool and with a custom ratio allow for retrieving selected target nuclear loci and, for example, full plastomes, mitochondrial-encoded protein-coding genes, or, depending on the sequencing depth, full mitochondrial chromosomes. Currently, three main universal enrichment kits exist for major land plant lineages, angiosperms: Angiosperms353 kit of Johnson et al. (2019) and the Angiosperm v.1 kit of Buddenhagen et al. (2016), current update is v.4; and flagellate land plant lineages and gymnosperms: GoFlag 451 and its optimized version GoFlag 408 (Breinholt et al. 2021). While these different universal enrichment kits work for angiosperms, ferns, gymnosperms, and lycophytes, they share a common set of target regions that allow for international collaborations, data reuse, and integration with other worldwide consortia such as the Kew Tree of Life (https://treeoflife.kew.org/#), GoFlag (https://flagellateplants.org/about-us/), and Australian Angiosperm Tree of Life (https://www.genomicsforaustralianplants.com/stage-1-aatol-eoi/). At the same time, the generated data remain open for integrating future sequence data and a plethora of already available genomic resources from public raw sequence data repositories from other individual genome skimming, Hyb-Seq, TE, and RNA-seq projects available through the Sequence Read Archive (https://www.ncbi.nlm.nih.gov/sra).

For many type genomics projects, the first-pass sequencing will represent the end of the line in terms of how much sequencing data can be generated at a reasonable cost considering the quality of the DNA (Fig. 2). To make a respective decision, we suggest a relatively simple quality control step to assess if further effort in the type sequencing is warranted. Average genome coverage, BUSCO representation, and plastome genome completeness are easy indicators to assess if the DNA may yield additional genomic information from a second pass of long-read sequencing approach.

### Second-Pass Sequencing—Maximizing the Type Genomic Information

**Figure 3.**
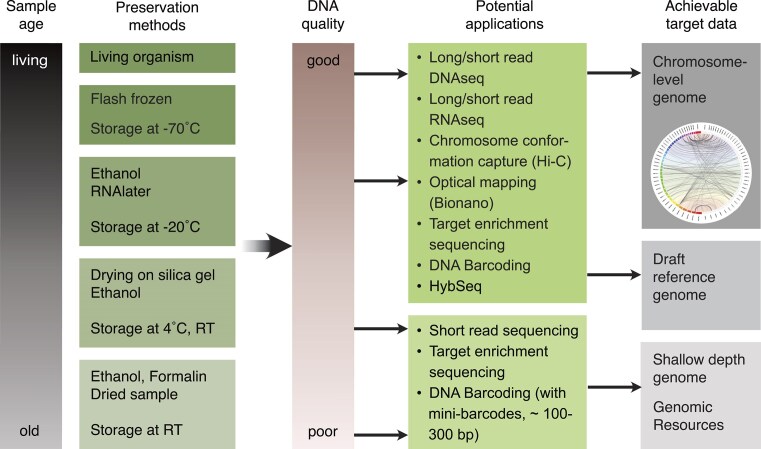
Overview of preservation methods and possible genetic/genomic sequencing applications with varying results in terms of quality/usability of target data. The longer the storage time, the less likely the different applications will work and the best target data quality will be achieved due to DNA degradation. This applies to every different preservation method. The colder the sample is stored, the better the DNA preservation, whereby it has been shown that −70 °C is just as effective as −80 °C. Jupiter plot from Nebenführ et al. (2024).

In some cases, DNA quality and yield and the results from the first-pass sequencing may indicate that a more complete genomic resource can be generated with additional financial and time effort. For example, type material that has been properly stored, newly collected, or can even be retrieved from living specimens can be used for long-read sequencing technologies such as PacBio and Oxford Nanopore. Additionally, scaffolding techniques such as High Throughput Chromosome Conformation Capture (Hi-C) chromatin interaction mapping, optical mapping, and linked-read sequencing are essential to achieve chromosome-level genomes (Fig. 3). These methods may be applied with sufficiently well-preserved samples and high DNA quality. However, it has been shown that suboptimally preserved samples (e.g., stored in 96% ethanol) are at least suitable for use in PacBio HiFi sequencing (Dahn et al. 2022; Bein et al. 2025). Recent advances in the preparation of ultra-low input DNA libraries (<5 ng total input DNA) even allow long-read sequencing of individual specimens of minute size, for example, single collembolans (Schneider et al. 2021; Roberts et al. 2024) or acoel flatworms (Abalde et al. 2023), demonstrating the success of genomics with even minimal input material.

While well-preserved DNA can be easily extracted in an average molecular laboratory by standard DNA preparation methods (Russell and Sambrook 2001), implementing a reference-quality genome sequencing approach may require a significant upfront investment in long-read sequencing and chromatin conformation capture. While this may present a financial challenge, high-quality genomes contribute directly to the global initiative of the Earth BioGenome Project (Lewin et al. 2022) by representing previously unknown and thus unaccounted species. Also, having already extracted DNA from a type specimen, a consortium should aim to maximize the genomic information output that can be generated in order to limit the future need for another potentially destructive sampling of the type specimen. The investment in funding high-quality genomes for type specimens has long-term implications for the permanent documentation of biodiversity.

### Preserving Future Type Specimens

The primary challenge for tissue preservation is to prevent enzymatic (or hydrolytic) degradation of nucleic acids, which can be minimized by cold storage and/or dehydration. For the former, the ideal method is flash-freezing of fresh samples followed by storage at −70 °C or in liquid nitrogen, ideally in frozen collections dedicated to the preservation of samples for genomics, such as biobanks (e.g., Corrales et al. 2023). In most cases, this allows the generation of a chromosome-level reference genome assembly. Dehydration methods include the storage of tissue samples in absolute ethanol (for DNA preservation in animals), a supersaturated salt solution (such as RNAlater for RNA preservation), or by drying with silica gel. These methods can preserve HMW DNA to some extent, and to an even greater extent if combined with cold storage.

Because any newly collected specimen may later potentially become a type specimen, investment in quality tissue preservation safeguards comprehensive genomic data for posterity. The realities of fieldwork mean that ideal preservation methods are not always possible, but all new field collections should include tissue preservation (or, where feasible, cell cultures) as part of the sampling protocol. For tissue preservation methods and their potentially applicable genomic sequencing methods, see Figure 2. Postspecimen collection tissue sampling is also possible at later stages of the accessioning process. For example, large amounts of tissue are removed during preparation of mammal and bird skins, which can be preserved for DNA/RNA sequencing given appropriate storage facilities such as dedicated cryocollections). Frozen tissue collections do not have the long history of traditional NHCs, and the long-term energy and storage implications are under continuous development. For now, permanent museums remain the “gold standard” as repositories for type material with or without cryocollection repositories.

Ideally, field collecting campaigns would include taxonomic, molecular biology, and cell culturing expertise, for example, through joint fieldwork or good standard operating procedures, and be sufficiently funded to maximize sampling success and ensure the proper preservation of both morphological and molecular specimen information. In remote or imperiled environments, such an interdisciplinary team could work together to improve the understanding of the local biodiversity, identify potential new species, ensure the appropriate storage of samples for future genomic analyses, and together extend the value of each specimen through the generation of high-quality and comprehensive biological data.

### Genomics of Type-Less Species or Species with Illustrations as Types

For animals, the ICZN (1999), in Article 73 and Declaration 45, allows the establishment of new species-group taxa without preserved name-bearing type material under special circumstances, albeit with caution. In these cases, authors are urged to compensate for the absence of physical type specimens with thorough documentation, including high-resolution images and DNA sequences, so that diagnostic characters are detailed as comprehensively as possible. This approach is particularly relevant for the study of soft-bodied meiofaunal organisms, where the deposition of physical type specimens is often impractical or inadvisable (Garraffoni et al. 2019). For taxonomic identification of these taxa, high-quality photomicrographs showcasing both internal and external diagnostic features are crucial. However, some specimens may be completely destroyed during preparation or storage, rendering them unsuitable for morphological studies or reducing them to mere clumps of tissue (Garraffoni et al. 2019). Nonetheless, this tissue can remain viable for genomic DNA extraction. Existing practices of species descriptions combining photomicrographs with DNA sequences underscore the importance of genetic data in taxonomy (e.g., Westheide and Hass-Cordes 2001; Kolicka et al. 2016; Kieneke and Nikoukar 2017). For a type-less species, the generation of genomic data and ensuring its access is not only highly recommended but is essential for the enduring definition of the species. For plants and fungi, the ICN (Turland et al. 2018), in Article 9.1 and 40.4, allows for illustrations as types of species described before 2007. Designation of an epitype (Art. 9.9, meant as an aid to interpret ambiguous or fragmentary types) is increasingly applied for cryptic species that turn up as a result of DNA-based taxonomy. If possible, it is advisable to select specimens as epitypes for which not only single DNA sequences but also genomic data are available. For microbial eukaryotes and prokaryotes, genomic resources might be achieved by single-cell transcriptome sequencing (e.g., Shazib et al. 2025 ).

## Unifying Taxonomy and Genomics to Unlock the Global Collection

The societal value of NHCs lies in their role as an archive of biodiversity through space and time. The accessibility of collections is critical to their value as research infrastructure and societal education resources. The digitization of specimen metadata and the adoption of the extended specimen concept with digitized phenotypic data (e.g., linear measurements, photographs, X-rays, computed tomograpy [CT] scans, spectrographic analysis), metadata such as call recordings (e.g., in frogs, birds, and certain arthropods) or environment (e.g., habitat or geology for plants), and molecular sequence information increase NHCs specimens’ values to both the scientific community and to society. While the analysis of hDNA requires expertise and specialized laboratory facilities (Fulton and Shapiro 2019), the inclusion of genetic and genomic information as part of an “extended type specimen” makes museum specimens generally accessible to a wide range of researchers and provides an easy way to share type information globally (cf. Davis et al. 2023).

Close and meaningful collaboration between curators, taxonomists, and genomics researchers can maximize the quality and quantity of data derived from type specimens. This requires effective communication, mutual investment, and sharing of benefits for success. A priori, sequencing of type material (old or future) is associated with apparently conflicting goals: preserving the morphological integrity of valuable type material by avoiding destructive sampling versus prioritizing the sampling of tissues in quantities that ensure usable amounts of DNA from potentially degraded biological material or supporting the testing of multiple samples of unknown quality. This conflict may put pressure on curators, as they are the temporary custodians of NHCs that also belong to future generations. Curators and taxonomic specialists are the experts who must decide which type specimens can and should be sampled, and which morphological structures are dispensable for taxonomic purposes and thus suitable for DNA extraction. Genomics researchers can provide expertise on the appropriate tissue type and the minimum amount needed for DNA extraction. For example, as much as 50 mg of tissue may be required in some cases, and a higher extraction success rate has been reported for internal organs such as the liver from formalin-fixed vertebrates (Hahn et al. 2022). Future developments in genomic methods may invalidate current guidelines and reduce the invasiveness of tissue sampling (Pätzold et al. 2020). Until then, new specimen collection efforts should consider sampling, fixation, and storage strategies to improve preservation of specimens and tissue samples that may one day become name-bearing types (Fig. 2).

Several scientific considerations and methodological innovations could pave the way for interdisciplinary collaborations, including the establishment of taxon-specific best practices for minimally destructive sampling and methods to screen specimens for genomic suitability (e.g., by assessing residual formalin concentration or tissue degradation; see Hahn et al. 2022). Further development of laboratory and computational infrastructure in natural history museums and herbaria would significantly improve the success of type sequencing efforts. The establishment of NHCs’ hDNA networks with in-house laboratories would make the movement of type specimens across institutions for museomics unnecessary, and allow in-house control over which specimens and structures are sampled for hDNA extraction. Wet lab protocols for hDNA extraction are material-dependent, but due to the low endogenous DNA content in many historical samples, they are prone to contamination with DNA and polymerase chain reaction (PCR) products from well-preserved tissue and DNA contaminants. Therefore, all work should be carried out in a contamination-free environment: ideally in a facility separated from the main laboratories, as developed for aDNA work (Rohland and Hofreiter 2007; Fulton and Shapiro 2019).

Museum-based hDNA extraction networks must go hand-in-hand with the development, testing, and standardization of hDNA extraction protocols. However, the development of such “museomics” facilities and hiring expert labor comes with a high economic cost. Because this may not be feasible at every institution, establishing collaborative networks among NHCs is necessary, and it provides opportunities for collaboration, training, and capacity building. Collaborations between more resource-rich institutions in the Global North and research institutions in the biodiversity-rich Global South may be particularly fruitful. As the majority of newly described species are likely to come from the Global South, the development of local laboratory infrastructure is ideal for faster and easier generation of genomic data. Within these networks, institutions in the Global North could economically support such initiatives and, in return, gain access to the genomic (and other digital) data without moving type specimens outside their country of origin.

Global museomic networks would also facilitate sharing of taxonomic expertise, which is sparsely distributed for a variety of taxonomic groups. Because taxonomists represent the main stakeholders for type material, any project seeking to sequence genomes from historical type material must first establish contact and discourse with the taxonomic specialist(s) for the group of interest. Individuals with curatorial responsibilities in NHCs may not have sufficient expertise with a given group to confidently advise on appropriate sampling strategies. Some curators may also be responsible for a wide range of organismal groups, so it may be necessary to consult specialists in the broader research community. Professional networks and scholarly societies could also facilitate the exchange of taxonomic expertise to optimize decisions on tissue sampling strategies in cases where such expertise is locally missing. Overall, prior communication with taxonomic specialists secures buy-in from experts whose research depends on the physical integrity of type specimens, allows sensible decision-making on which types to sample and how, and balances physical information loss against genomic sequence information gain. Collaborating taxonomists may also choose to fix the definition of a species to a single sequenced specimen, if appropriate, for example, by lectotype designation from a syntype series (Fig. 1b).

The potential benefits for NHCs and the scientific community are enormous. Increasing the scientific value of collections while minimizing the risk of damage and loss of type specimens solidifies NHCs as important research resources. Type genomics benefits taxonomy by providing powerful, additional lines of evidence for species characterization. By uniting taxonomy and genomics, we modernize NHCs through holistic and open-access specimen information with genomic data, high-quality images, geographic information, morphological data, etc., which can be made freely available globally through public databases and data structures (e.g., Global Biodiversity Information Facility [GBIF], Barcode of Life Databas [BOLD], European Molecular Biology Laboratory [EMBL], and GenBank).

## Outlook

The name-bearing type specimens provide the physical link between a representative specimen and the scientific name of a species. Protecting the integrity of these specimens while ensuring broad access to the information they hold to the scientific community will be a central challenge for NHCs in the coming decades. We propose that a fundamental part of a type specimen's biological information, its genome, can be made freely and digitally available through *type genomics*. Sequencing genomes of name-bearing types allows a wide range of genetic characterizations and provides a perpetual digital reference. Open availability of this digital data enables a plethora of downstream genetic analyses without further physical damage to the type specimen, and provides access to the global research community (Renner et al. 2024). Importantly, a “low coverage” genomic resource is often feasible and completely sufficient to genetically characterize the type specimen in the context of taxonomic and genomic comparison with conspecific or other specimens in many areas of biodiversity research.

Type genomics is likely to become a central pillar of digitization for modern NHCs. Natural history museums and herbaria are increasingly providing access to digital collections. The vast size of many of these collections makes comprehensive and complete specimen digitization a distant, yet achievable, goal. A first focus on type specimens has several key benefits. Types, particularly name-bearing types, are the most sought-after specimens for scientists seeking to access collections and their data. The creation of a “digital twin” through holistic-type digitization reduces the physical risk to these specimens from repeated handling and loans. Researchers can access type specimen information easily and more or less instantaneously. Therefore, providing open genomic characterizations of type specimens enhances the quality, research utility, and access of type specimen data while preserving the integrity of these historically significant specimens.

To truly embrace and harness the power of type genomics, we should build collaborative, interdisciplinary networks and infrastructure with particular attention to collaborations with local researchers at fieldwork sites. This would enable mutually beneficial, cost-effective laboratory work; facilitate field logistics and permitting; and provide an opportunity for meaningful benefit sharing through the generation of open and freely accessible specimen data. NHCs, taxonomists, and experts in genomic methods can and should work together to develop open and modern biodiversity research worldwide.

## Supplementary Material

syaf040_Supplemental_File Data available from the Dryad Digital Repository: http://dx.doi.org/10.5061/dryad.zs7h44jn5
