# Supplementary material for: Type Genomics: A Framework for Integrating Genomic Data into Biodiversity and Taxonomic Research
Source: Syst Biol. 2025 May 20;74(6):1029–44. doi: 10.1093/sysbio/syaf040 (PMC12712332; doi:10.1093/sysbio/syaf040)
Supplement: syaf040_Supplemental_File — Data available from the Dryad Digital Repository: http://dx.doi.org/10.5061/dryad.zs7h44jn5 [file syaf040_supplemental_file.pdf]

Supplement

Table S1: hDNA and aDNA extraction protocols used for type specimens

a) Bones, teeth: Protocols must take into account that the DNA is enclosed in hard calcified cavities.

**Bones, teeth, shells**	**Advantages**	**Disadvantages**	**Examples of type specimen studies**
Pioneering ancient DNA protocols with decontamination & decalcification of bone, guanidinium thiocyanate extraction with phenol-chloroform purification (Russell and Sambrook 2001) and silica-in-solution purification (Höss and Pääbo 1993)	manual, inexpensive	Complex, time-consuming manual work	Krings et al.(1997; Mammalia), adapted for tissue by Kirchmann et al. (2009; Aves), Hofreiter et al. (2001; Mammalia), Dalebout et al. (2014; Mammalia)
Different, specific extraction buffers, purification using GuSCN/silica suspension by Rohland et al. (2004), Hofreiter et al. (2004)	manual, inexpensive	Complex, time-consuming manual work	Scherz et al. (2020; Amphibia)
Early protocol combining decontamination & decalcification of bone with DNeasy Tissue Kit (Qiagen), Kearney & Stuart (2004)	silica-column based kit combination saves time	small DNA fragments are not retained	Stuart and Fritz (2008; Testudines)
Silica-in-solution protocol to reduce further DNA damage, Rohland and Hofreiter (2007)	scalable, PCR inhibitors removed	Requires substantial time for buffer & silica suspension preparation	Olsen et al. (2016; Mammalia), Li et al. (2015; Pisces)
Silica-based column, Rohland et al. (2010)	faster than in-solution silica protocol	Single company producing specialised columns	Li et al. (2015; Pisces)
Subfossil bone protocol targeting fragmented, short single-stranded DNA via modified use of a MinElute silica column in a custom-built apparatus, Dabney et al. (2013)	DNA yield of short fragments is much improved	Complex manual preparation, expensive custom-built apparatus	Kehlmaier et al. (2017; Testudines), Scherz et al. 2020; Amphibia), Vershinina et al. (2020; Mammalia), Kehlmaier et al. (2023; Testudines)

b) Tissue, wet and dry: DNA extraction of tissue from museum specimens (plants, fungi and animals) has a much more diverse set of approaches.

**Wet tissue**	**Advantages**	**Disadvantages**	**Examples of type specimen studies**
Dabney et al. (2013) Extraction protocol adapted for tissue.	Improved DNA yield of short fragments	Complex and time-consuming manual work, expensive custom-built apparatus	McGuire et al. (2018; Lepidosauria), Rancilhac et al. (2020; Amphibia), Boubli et al. (2018; Mammalia)
Dabney et al. Extraction protocol adapted for tissue with proteinase-K lysis from Sambrook & Russell (2001) by Straube et al. (2021a)	Improved DNA yield of short fragments	Complex and time-consuming manual work, expensive custom-built apparatus	Straube et al. (2021b; Pisces), Vences et al. (2021; Amphibia), Agne et al. (2022; Pisces)
PacBio ultra-low library protocol adapted with alternative whole genome amplification polymerase, Bein et al. (2025)	Minimal DNA input with high sequencing performance, reduced PCR bias with alternative whole genome amplification polymerase.	Whole genome amplification step introduces risk of PCR bias.	none yet
Phenol/chloroform extraction for well-preserved frozen tissue in ethanol	Well-proven and de facto standard method for high-quality genomes	Irritating chemicals	Schöneberg et al. (2023; Lepidosauria)
Sera-Mag library cleanup SpeedBeads adapted for DNA extraction, Rázuri-Gonzales et al. (2022)	Inexpensive	Tricky pipetting, requires magnetic apparatus	Mahony et al. (2022; Amphibia), Heckenhauer et al. (2023; Insecta)
Hot alkaline lysis gDNA extraction from formalin-fixed archival tissues (V.2), Hahn et al. (2022)	Good DNA yield, robust protocol, good potential for difficult samples	Low-throughput, requires specific devices, 2-day protocol	Hahn et al. (2022)
**Dry tissue**	**Advantages**	**Disadvantages**	**Examples of type specimen studies**
CTAB extraction protocol for plants, Doyle and Doyle (1987) by Rogers and Bendich (1985) and partly adapted by Porebski et al. (1997)	Well-proven	Toxic chemicals, complex and time-consuming manual work	Larsson and Jacobsson (2004; Basidiomycota), Andreasen et al. (2009; Streptophyta), Hughey and Gabrielson (2012; Rhodophyta), Hosaka and Uno (2013; Basidiomycota), Erpenbeck et al. (2016; Porifera), Richards et al. (2021b; Rhodophyta), Contreras-Ortiz et al. (2019; Angiosperms), Hughey et al. (2019; Chlorophyta), Richards et al. (2021a; Rhodophyta)

Mercaptoethanol extraction buffer, Dellaporta et al. (1983), followed by chloroform extraction step and DNA precipitation, Hughey et al. (2001)	Well-proven	Toxic chemicals, complex and time-consuming manual work	(Rhodophyta: Hughey et al. 2002, 2014; Gabrielson et al. 2011; Hind et al. 2014; Mateo-Cid et al. 2014; Hernandez-Kantun et al. 2015; Maneveldt et al. 2020; Calderon et al. 2021; Peña et al. 2021; Richards et al. 2021a; Schipper et al. 2023; Wade et al. 2023) (Chlorophyta: Hughey et al. 2024)
DNAzol reagent (Gibco BRL/Life Technologies, Gaithersburg, MD, U.S.A.) combined with chaotropic agent guanidine thiocyanate and detergent mixture for lysis, permits selective precipitation of DNA from a cell lysate by Junqueira et al. (2002)	Best-performing of three tested methods, better performance than chelex and phenol/chloroform for old specimens	Toxic chemicals, complex and time-consuming manual work	Maddison and Cooper (2014; Insecta)
Immersion of entire beetles followed by phenol/chloroform (Sambrook et al. 1989) extraction by Gilbert et al. (2007)	Minimally destructive, well-proven	Toxic chemicals, complex and time-consuming manual work	Maddison and Cooper (2014; Insecta)
Silica-based glass fibre filtration (GF) plates in 96-well format for automation, (Ivanova et al. 2006; Dewaard et al. 2008)	Inexpensive, automation-friendly	Targets HMW DNA/long fragments, degraded small fragments lost	(Insecta: Vaglia et al. 2008; Rougerie et al. 2012; Mutanen et al. 2015; Speidel et al. 2015; Prosser et al. 2016; Hausmann et al. 2017)
Mechanical disruption of spores using single acid-washed glass bead (Frommlet and Iglesias-Rodríguez 2008)	Minimally destructive, no DNA cleanup, direct to PCR	Delicate procedure	Janik et al. (2020; Myxomycetes)
sbeadex forensic kit (LGC), Hundsdoerfer and Kitching (2010)	Purifies DNA of different sizes using magnetic beads, adaptable for formalin-fixed wet tissue	Special magnet equipment needed, tricky pipetting	Stuckas and Fritz (2021; Lepidosauria, formalin-fixed), Mende and Hundsdoerfer (2013; Insecta), Fritz et al. (2014; Testudines), Petzold et al. (2014; Testudines), Hundsdoerfer et al.(2017; Insecta), Hundsdoerfer and Kitching (2017; Insecta), Kehlmaier et al. (2020; Lepidosauria, formalin-fixed), Schweizer et al. (2020; Aves)
Chelex X-100 beads (BioRad) (Junqueira et al. 2002)	Cheap	DNA is not purified	Shumskaya et al. (2023; Basidiomycota), Ferencova et al. (2017, lichens)

E.Z.N.A. Plant DNA DS Mini Kit (Omega Biotek) (Kistenich et al. 2019)	Fast, easy, well-proven	Targets HMW DNA/long fragments, degraded small fragments lost	Leavitt et al. (2019; lichen)
Oligo-nucleotide and PCR-cleanup kit (NEB) (Patzold et al. 2020),<br><br>Qiagen QIAquick PCR purification kit (Knyshov et al. 2019a)	Fast, targets both small/degraded DNA fragments and large fragments	Special magnet equipment needed, tricky pipetting	Patzold et al. (2021; Insecta), Knyshov et al. (2019b; Insecta)
GEN-IAL All-tissue DNA-Kit (GEN-IAL GmbH, Troisdorf, Germany)	Cheap, universally applicable	Somewhat time-consuming	(Stelbrink et al. 2019; Mollusca), Wilke et al. (2023; Mollusca)
QIAGEN DNA isolation kits (e.g., DNeasy Blood & Tissue kit, Genomic-tip 20/G, QIAamp DNA Investigator Kit, QiAmp Mini Kit, QIAamp DNA Micro Kit) (Qiagen, West Sussex, UK or Hilden, Germany) used for dry plant, fungal and animal tissue	Fast, easy, well-proven	Expensive, targets HMW DNA/long fragments, but can also work well for smaller fragments	Ronikier et al. (2022; Myxomycetes, Amoebozoa), Ernst et al. (2021; Amphibia), Kirschel et al. (2018; Aves), Collinson et al. (2018; Aves), Shumskaya et al. (2023; Basidiomycota), Taylor et al. (2017; Chlorophyta), Puillandre et al. (2011; Crustacea), (Insecta: Strutzenberger et al. 2012; Mammalia: Cappellini et al. 2014; Price et al. 2015; Mantellatto et al. 2021; Mayer et al. 2021; Twort et al. 2021; Castañeda-Rico et al. 2022), Vieira et al. (2016; Ochrophyta), (Pisces: Silva et al. 2019; Sullivan et al. 2022), Erpenbeck et al. (2016; Porifera), (Testudines: Praschag et al. 2008; Kehlmaier et al. 2019), (Rhodophyta: Hernandez-Kantun et al. 2015, 2016; Suzuki et al. 2016; Peña et al. 2021; Gabrielson et al. 2023a, 2023b)
QiaQuick PCR purification kit (Qiagen, West Sussex, UK or Hilden, Germany)	Fast, targets both small/degraded DNA fragments and large fragments	Expensive	Kirschel et al. (2018; Aves)
NucleoSpin Plant II Kit (Macherey-Nagel, Düren, Germany)	Fast, easy, well-proven	Targets HMW DNA/long fragments, degraded small fragments lost	Hausmann et al. (2009; Insecta), Chomicki and Renner (2015; Angiosperms), Jeong et al. (2019; Rhodophyta), Maneveldt et al. (2020; Rhodophyta)

QuickExtract Plant DNA Extraction Solution (Epicentre, Madison, WI, USA)	Extremely fast, simple, non-toxic reagents, no mechanical procedures or centrifugation needed	DNA is not purified	Hanyuda and Kawi (2018; Chlorophyta)
Sorbitol washing complex homogenate for improved DNA extractions (Jones et al. 2021)	Removal of polyphenols and polysaccharides in plant and fungi samples	Toxic chemicals	none yet

References

Agne S., Naylor G.J., Preick M., Yang L., Thiel R., Weigmann S., Straube N. 2022. Taxonomic identification of two poorly known lantern shark species based on mitochondrial DNA from wet-collection paratypes. Front. Ecol. Evol. 10:910009.

Andreasen K., Manktelow M., Razafimandimbison S.G. 2009. Successful DNA amplification of a more than 200-year-old herbarium specimen: recovering genetic material from the Linnaean era. TAXON. 58:959–962.

Bein B., Chrysostomakis I., Arantes L.S., Brown T., Gerheim C., Schell T., Schneider C., Leushkin E., Chen Z., Sigwart J., Gonzalez V., Wong N.L.W.S., Santos F.R., Blom M.P.K., Mayer F., Mazzoni C.J., Böhne A., Winkler S., Greve C., Hiller M. 2025. Long-read sequencing and genome assembly of natural history collection samples and challenging specimens. Genome Biol. 26:25

Boubli J.P., da Silva M.N.F., Rylands A.B., Nash S.D., Bertuol F., Nunes M., Mittermeier R.A., Byrne H., Silva F.E., Röhe F., Sampaio I., Schneider H., Farias I.P., Hrbek T. 2018. How many pygmy marmoset (*Cebuella* Gray, 1870) species are there? A taxonomic re-appraisal based on new molecular evidence. Mol. Phylogenet. Evol. 120:170–182.

Calderon M.S., Bustamante D.E., Gabrielson P.W., Martone P.T., Hind K.R., Schipper S.R., Mansilla A. 2021. Type specimen sequencing, multilocus analyses, and species delimitation methods recognize the cosmopolitan *Corallina berteroi* and establish the northern Japanese *C. yendoi* sp. nov. (Corallinaceae, Rhodophyta). J. Phycol. 57:1659–1672.

Cappellini E., Gentry A., Palkopoulou E., Ishida Y., Cram D., Roos A.M., Gilbert M.T.P. 2014. Resolution of the type material of the Asian elephant, *Elephas maximus* Linnaeus, 1758 (Proboscidea, Elephantidae. Zool. J. Linn. Soc. 170:222–232.

Castañeda-Rico S., Edwards C.W., Hawkins M.T., Maldonado J.E. 2022. Museomics and the holotype of a critically endangered cricetid rodent provide key evidence of an undescribed genus. Front. Ecol. Evol. 10:930356.

Chomicki G., Renner S.S. 2015. Watermelon origin solved with molecular phylogenetics including Linnaean material: another example of museomics. New Phytol. 205:526–532.

Collinson J.M., Päckert M., Lawrie Y., Gatter W., Töpfer T., Phalan B., Fishpool L. 2018. Taxonomic status of the Liberian Greenbul *Phyllastrephus leucolepis*. J. Orn. 159:19–27.

Contreras-Ortiz N., Rodríguez-García T., Quintanilla S., Bernal-Villegas J., Madriñán S., Gómez-Gutiérrez A. 2019. The origin of Humboldt and Bonpland's holotype of *Oncidium ornithorhynchum*, clarified using +200-year-old DNA. Taxon. 68:471–480.

Dabney J., Knapp M., Glocke I., Gansauge M.T., Weihmann A., Nickel B., Meyer M. 2013. Complete mitochondrial genome sequence of a Middle Pleistocene cave bear reconstructed from ultrashort DNA fragments. Proc. Natl. Acad. Sci. 110:15758–15763.

Dalebout M.L., Scott Baker C., Steel D., Thompson K., Robertson K.M., Chivers S.J., Yamada T.K. 2014. Resurrection of *Mesoplodon hotaula* Deraniyagala 1963: A new species of beaked whale in the tropical Indo-Pacific. Mar. Mammal Sci. 30:1081–1108.

Dellaporta S.L., Wood J., Hicks J.B. 1983. A plant DNA mini-preparation: Version II. Plant Molec Biol Rep. 1:19–21.

Dewaard J.R., Ivanova N.V., Hajibabaei M., Hebert P.D.N. 2008. Assembling DNA barcodes: analytical protocols. In: Martin C., editor. Methods in molecular biology: environmental Genetic data. Totowa, New Jersey: Humana Press. p. 275–293.

Doyle J.J., Doyle J.L. 1987. A rapid DNA isolation procedure for small quantities of fresh leaf tissue. .

Ernst R., Kehlmaier C., Baptista N.L., Pinto P.V., Branquima M.F., Dewynter M., Schmitz A. 2021. Filling the gaps: The mitogenomes of Afrotropical egg-guarding frogs based on historical type material and a re-assessment of the nomenclatural status of *Alexteroon* Perret, 1988 (Hyperoliidae. Zool. Anz. 293:215–224.

Erpenbeck D., Ekins M., Enghuber N., Hooper J.N.A., Lehnert H., Poliseno A., Schuster A., Setiawan E., Voogd N.J.D., Wörheide G., Soest R.W.M.V. 2016. Nothing in (sponge) biology makes sense – except when based on holotypes. J. Mar. Biol. Assoc. U. K. 96:305–311.

Ferencova Z., Rico V.J., Hawksworth D.L. 2017. Extraction of DNA from lichen-forming and lichenicolous fungi: a low-cost fast protocol using Chelex. Lichenologist. 49:521-525.

Fritz U., Petzold A., Kehlmaier C., Kindler C., Campbell P., Hofmeyr M.D., Branch W.R. 2014. Disentangling the *Pelomedusa* complex using type specimens and historical DNA (Testudines: Pelomedusidae. Zootaxa. 3795:501–522.

Frommlet J.C., Iglesias-Rodríguez M.D. 2008. Microsatellite genotyping of single cells of the dinoflagellate species *Lingulodinium polyedrum* (Dinophyceae): A novel approach for marine microbial population genetic studies[1]. *J. Phycol.* 44:1116–1125.

Gabrielson P.W., Hughey J.R., Peña V., Richards J.L., Saunders G.W., Twist B., Nelson W.A. 2023a. Asia Pacific *Sporolithon* (Corallinophycidae, Rhodophyta) species revised based on DNA sequencing of type specimens and including *S. crypticum* sp. nov. Phycol. 62:593–607.

Gabrielson P.W., Maneveldt G.W., Hughey J.R., Peña V. 2023b. Taxonomic contributions to Hapalidiales (Corallinophycidae, Rhodophyta): *Boreolithothamnion* gen. nov., *Lithothamnion* redefined and with three new species and *Roseolithon* with new combinations. J. Phycol. 59:751–774.

Gabrielson P.W., Miller K.A., Martone P.T. 2011. Morphometric and molecular analyses confirm two distinct species of *Calliarthron* (Corallinales, Rhodophyta), a genus endemic to the northeast Pacific. Phycologia. 50:298–316.

Gilbert M.T.P., Moore W., Melchior L., Worobey M. 2007. DNA Extraction from Dry Museum Beetles without Conferring External Morphological Damage. PLOS ONE. 2:e272.

Hahn E.E., Alexander M.R., Grealy A., Stiller J., Gardiner D.M., Holleley C.E. 2022. Unlocking inaccessible historical genomes preserved in formalin. Mol. Ecol. Resour. 22:2130–2147.

Hanyuda T., Kawai H. 2018. Genetic examination of the type specimen of *Ulva australis* suggests that it was introduced to Australia. Phycol. Res. 66:238–241.

Hausmann A., Hebert P.D., Mitchell A., Rougerie R., Sommerer M., Edwards T., Young C.J. 2009. Revision of the Australian *Oenochroma vinaria* Guenée, 1858 species-complex (Lepidoptera: Geometridae, Oenochrominae): DNA barcoding reveals cryptic diversity and assesses status of type specimen without dissection. Zootaxa. 2239:1–21.

Hausmann A., Miller S.E., Holloway J.D., deWaard J.R., Pollock D., Prosser S.W.J., Hebert P.D.N. 2017. Calibrating the taxonomy of a megadiverse insect family: 3000 DNA barcodes from geometrid type specimens (Lepidoptera, Geometridae). 6th Int. Barcode Life Conf. 01:671–684.

Heckenhauer J., Razuri-Gonzales E., Mwangi F.N., Schneider J., Pauls S.U. 2023. Holotype sequencing of *Silvatares holzenthali* Rázuri-Gonzales, Ngera & Pauls, 2022 (Trichoptera, Pisuliidae). ZooKeys. 1159:1–15.

Hernandez-Kantun J.J., Gabrielson P., Hughey J.R., Pezzolesi L., Rindi F., Robinson N.M., Adey W. 2016. Reassessment of branched *Lithophyllum* spp.(Corallinales, Rhodophyta) in the Caribbean Sea with global implications. Phycologia. 55:619–639.

Hernandez-Kantun J.J., Rindi F., Adey W.H., Heesch S., Peña V., Le Gall L., Gabrielson P.W. 2015. Sequencing type material resolves the identity and distribution of the generitype *Lithophyllum incrustans,* and related European species *L. hibernicum* and *L. bathyporum* (Corallinales, Rhodophyta). J. Phycol. 51:791–807.

Hind K.R., Gabrielson P.W., Lindstrom S.C., Martone P.T. 2014. Misleading morphologies and the importance of sequencing type specimens for resolving coralline taxonomy (Corallinales, Rhodophyta): *Pachyarthron cretaceum* is *Corallina officinalis*. J. Phycol. 50:760–764.

Hofreiter M., Rabeder G., Jaenicke-Després V., Withalm G., Nagel D., Paunovic M., Pääbo S. 2004. Evidence for reproductive isolation between cave bear populations. Curr. Biol. 14:40–43.

Hofreiter M., Serre D., Poinar H.N., Kuch M., Pääbo S. 2001. Ancient DNA. Nat. Rev. Genet. 2:353–359.

Hosaka K., Uno K. 2013. Assessment of the DNA quality in mushroom specimens: a recovery of the whole ITS sequence from fragmented DNA of the type specimen. Bull. Natl. Mus. Nat. Sci. Ser. B Bot. 39:53–60.

Höss M., Pääbo S. 1993. DNA extraction from Pleistocene bones by a silica-based purification method. Nucleic Acids Res.:21,3913-3914.

Hughey J., Silva P., Hommersand M. 2002. ITS1 sequences of type specimens of *Gigartina* and *Sarcothalia* and their significance for the classification of South African Gigartinaceae (Gigartinales, Rhodophyta). Eur. J. Phycol. 37:209–216.

Hughey J.R., Gabrielson P.W. 2012. Comment on “Acquiring DNA sequence data from dried archival red algae (Florideophyceae) for the purpose of applying available names to contemporary genetic species: a critical assessment.” Botany. 90:1191–1194.

Hughey J.R., Gabrielson P.W., Rohmer L., Tortolani J., Silva M., Miller K.A., Young J.D., Martell C., Ruediger E. 2014. Minimally destructive sampling of type specimens of *Pyropia* (Bangiales, Rhodophyta) recovers complete plastid and mitochondrial genomes. Sci. Rep. 4:5113.

Hughey J.R., Maggs C.A., Mineur F., Jarvis C., Miller K.A., Shabaka S.H., Gabrielson P.W. 2019. Genetic analysis of the Linnaean *Ulva lactuca* (Ulvales, Chlorophyta) holotype and related type specimens reveals name misapplications, unexpected origins, and new synonymies. J. Phycol. 55:503–508.

Hughey J.R., Miller K.A., Gabrielson P.W. 2024. Genetic analysis of *Ulva* (Ulvaceae, Chlorophyta) type specimens resolves northeast Pacific blade-forming species. Bot. Mar. 67:165–179.

Hughey J.R., Silva P.C., Hommersand M.H. 2001. Solving Taxonomic and Nomenclatural Problems in Pacific Gigartinaceae (rhodophyta) Using Dna from Type Material. J. Phycol. 37:1091–1109.

Hundsdoerfer A., Kitching I. 2010. A method for improving DNA yield from older specimens of large Lepidoptera while minimizing damage to external and internal abdominal characters. Arthropod Syst. Phylogeny. 68:151–155.

Hundsdoerfer A.K., Kitching I.J. 2017. Historic DNA for taxonomy and conservation: A case-study of a century-old Hawaiian hawkmoth type (Lepidoptera: Sphingidae). PloS One. 12:0173255.

Hundsdoerfer A.K., Päckert M., Kehlmaier C., Strutzenberger P., Kitching I.J. 2017. Museum archives revisited: Central Asiatic hawkmoths reveal exceptionally high late Pliocene species diversification (Lepidoptera, Sphingidae). Zool. Scr. 46:552–570.

Ivanova N.V., Dewaard J.R., Hebert P.D. 2006. An inexpensive, automation-friendly protocol for recovering high-quality DNA. Mol. Ecol. Notes. 6:998–1002.

Janik P., Ronikier M., Ronikier A. 2020. New protocol for successful isolation and amplification of DNA from exiguous fractions of specimens: a tool to overcome the basic obstacle in molecular analyses of myxomycetes. PeerJ. 8:8406.

Jeong S.Y., Won B.Y., Hassel K., Cho T.O. 2019. Revision of *Phymatolithon purpureum* (Hapalidiales, Rhodophyta) based on ultrastructural and molecular data. Eur. J. Phycol. 54:326–341.

Jones A., Torkel C., Stanley D., Nasim J., Borevitz J., Schwessinger B. 2021. High-molecular weight DNA extraction, clean-up and size selection for long-read sequencing. PLOS ONE. 16:e0253830.

Junqueira A.C.M., Lessinger A.C., Azeredo-Espin A.M.L. 2002. Methods for the recovery of mitochondrial DNA sequences from museum specimens of myiasis-causing flies. Med. Vet. Entomol. 16:39–45.

Kearney M., Stuart B.L. 2004. Repeated evolution of limblessness and digging heads in worm lizards revealed by DNA from old bones. Proc. R. Soc. Lond. B Biol. Sci. 271:1677–1683.

Kehlmaier C., Barlow A., Hastings A.K., Vamberger M., Paijmans J.L.A., Steadman D.W., Albury N.A., Franz R., Hofreiter M., Fritz U. 2017. Tropical ancient DNA reveals relationships of the extinct Bahamian giant tortoise *Chelonoidis alburyorum*. Proc. R. Soc. B Biol. Sci. 284:20162235.

Kehlmaier C., Graciá E., Ali J.R., Campbell P.D., Chapman S.D., Deepak V., Ihlow F., Jalil N.-E., Pierre-Huyet L., Samonds K.E., Vences M., Fritz U. 2023. Ancient DNA elucidates the lost world of western Indian Ocean giant tortoises and reveals a new extinct species from Madagascar. Sci. Adv. 9:eabq2574.

Kehlmaier C., López-Jurado L.F., Hernández-Acosta N., Mateo-Miras A., Fritz U. 2021. Ancient DNA reveals that the scientific name for an extinct tortoise from Cape Verde refers to an extant South American species. Sci. Rep. 11:17537.

Kehlmaier C., Zinenko O., Fritz U. 2019. The enigmatic Crimean green lizard (*Lacerta viridis magnifica*) is extinct but not valid: Mitogenomics of a 120-year-old museum specimen reveals historical introduction. J. Zool. Syst. Evol. Res. 58:303–307.

Kirchman J.J., Witt C.C., McGuire J.A., Graves G.R. 2009. DNA from a 100-year-old holotype confirms the validity of a potentially extinct hummingbird species. Biol. Lett. 6:112–115.

Kirschel A.N., Nwankwo E.C., Gonzalez J.C.T. 2018. Investigation of the status of the enigmatic White-chested Tinkerbird *Pogoniulus makawai* using molecular analysis of the type specimen. Ibis. 160:673–680.

Kistenich S., Halvorsen R., Schrøder-Nielsen A., Thorbek L., Timdal E., Bendiksby M. 2019. DNA sequencing historical lichen specimens. Front. Ecol. Evol. 7:5.

Knyshov A., Gordon E.R., Weirauch C. 2019a. Cost-efficient high throughput capture of museum arthropod specimen DNA using PCR-generated baits. Methods Ecol. Evol. 10:841–852.

Knyshov A., Hoey-Chamberlain R., Weirauch C. 2019b. Hybrid enrichment of poorly preserved museum specimens refines homology hypotheses in a group of minute litter bugs (Hemiptera: Dipsocoromorpha: Schizopteridae). Syst. Entomol. 44:985–995.

Krings M., Stone A., Schmitz R.W., Krainitzki H., Stoneking M., Pääbo S. 1997. Neandertal DNA sequences and the origin of modern humans. cell. 90:19–30.

Larsson E., Jacobsson S. 2004. Controversy over *Hygrophorus cossus* settled using ITS sequence data from 200 year-old type material. Mycol. Res. 108:781–786.

Leavitt S.D., Kueler R., Newberry C.C., Rosentreter R., Clair L.L. 2019. Shotgun sequencing decades-old lichen specimens to resolve phylogenomic placement of type material. Plant Fungal Syst.

Li C., Corrigan S., Yang L., Straube N., Harris M., Hofreiter M., White W.T., Naylor G.J.P. 2015. DNA capture reveals transoceanic gene flow in endangered river sharks. Proc. Natl. Acad. Sci. 112:13302–13307.

Maddison D.R., Cooper K.W. 2014. Species delimitation in the ground beetle subgenus *Liocosmius* (Coleoptera: Carabidae: *Bembidion*), including standard and next-generation sequencing of museum specimens. Zool. J. Linn. Soc. 172:741–770.

Mahony S., Nidup T., Streicher J.W., Teeling E.C., Kamei R.G. 2022. A review of torrent frogs (*Amolops*: Ranidae) from Bhutan, the description of a new species, and reassessment of the taxonomic validity of some *A. viridimaculatus* group species aided by archival DNA sequences of century-old type specimens. Herpetol. J. 32:142–175.

Maneveldt G.W., Jeong S.Y., Cho T.O., Hughey J.R., Gabrielson P.W. 2020. Reassessment of misapplied names, *Phymatolithon ferox* and *P. repandum* (Hapalidiales, Corallinophycidae, Rhodophyta) in South Africa, based on DNA sequencing of type and recently collected material. Phycologia. 59:449–455.

Mantellatto A.M.B., González S., Duarte J.M.B. 2021. Cytochrome b sequence of the *Mazama americana jucunda* Thomas, 1913 holotype reveals *Mazama bororo* Duarte, 1996 as its junior synonym. Genet. Mol. Biol. 45:20210093.

Mateo-Cid L.E., González A.C.M., Gabrielson P.W. 2014. *Neogoniolithon* (Corallinales, Rhodophyta) on the Atlantic coast of Mexico, including *N. siankanensis* sp. nov. Phytotaxa. 190:64–93.

Mayer C., Dietz L., Call E., Kukowka S., Martin S., Espeland M. 2021. Adding leaves to the Lepidoptera tree: capturing hundreds of nuclear genes from old museum specimens. Syst. Entomol. 46:649–671.

McGuire J.A., Cotoras D.D., O'Connell B., Lawalata S.Z.S., Wang-Claypool C.Y., Stubbs A., Huang X., Wogan G.O.U., Hykin S.M., Reilly S.B., Bi K., Riyanto A., Arida E., Smith L.L., Milne H., Streicher J.W., Iskandar D.T. 2018. Squeezing water from a stone: high-throughput sequencing from a 145-year old holotype resolves (barely) a cryptic species problem in flying lizards. PeerJ. 6:e4470.

Mende M.B., Hundsdoerfer A.K. 2013. Mitochondrial lineage sorting in action–historical biogeography of the *Hyles euphorbiae* complex (Sphingidae, Lepidoptera) in Italy. BMC Evol. Biol. 13:1–13.

Mutanen M., Kekkonen M., Prosser S.W., Hebert P.D., Kaila L. 2015. One species in eight: DNA barcodes from type specimens resolve a taxonomic quagmire. Mol. Ecol. Resour. 15:967–984.

Olsen M.T., Galatius A., Biard V., Gregersen K., Kinze C.C. 2016. The forgotten type specimen of the grey seal [*Halichoerus grypus* (Fabricius, 1791)] from the island of Amager, Denmark. Zool. J. Linn. Soc. 178:713–720.

Pätzold F., Marabuto E., Daneck H., O'Neill M.A., Kitching I.J., Hundsdoerfer A.K. 2021. The Phylogenetics and Biogeography of the Central Asian Hawkmoths, *Hyles hippophaes* and *H. chamyla:* Diversity. 13:213.

Pätzold F., Zilli A., Hundsdoerfer A.K. 2020. Advantages of an easy-to-use DNA extraction method for minimal-destructive analysis of collection specimens. PLOS ONE. 15:e0235222.

Peña V., Bélanger D., Gagnon P., Richards J.L., Le Gall L., Hughey J.R., Saunders G.W., Lindstrom S.C., Rinde E., Husa V., Christie H., Fredriksen S., Hall-Spencer J.M., Steneck R.S., Schoenrock K.M., Gitmark J., Grefsrud E.S., Anglès d'Auriac M.B., Legrand E., Grall J., Mumford T.F., Kamenos N.A., Gabrielson P.W. 2021. *Lithothamnion* (Hapalidiales, Rhodophyta) in the changing Arctic and Subarctic: DNA sequencing of type and recent specimens provides a systematics foundation. Eur. J. Phycol. 56:468–493.

Petzold A., Vargas-Ramirez M., Kehlmaier C., Vamberger M., Branch W.R., Preez L., Fritz U. 2014. A revision of African helmeted terrapins (Testudines: Pelomedusidae: *Pelomedusa*), with descriptions of six new species. Zootaxa. 3795:523–548.

Porebski S., Bailey L.G., Baum B.R. 1997. Modification of a CTAB DNA extraction protocol for plants containing high polysaccharide and polyphenol components. Plant Mol. Biol. Report. 15:8–15.

Praschag P., Sommer R.S., Mccarthy C., Gemel R., Fritz U. 2008. Naming one of the world's rarest chelonians, the southern Batagur. Zootaxa. 1758:61–68.

Price B.W., Henry C.S., Hall A.C., Mochizuki A., Duelli P., Brooks S.J. 2015. Singing from the grave: DNA from a 180 year old type specimen confirms the identity of *Chrysoperla carnea* (Stephens). PloS ONE. 10:0121127.

Prosser S.W., Dewaard J.R., Miller S.E., Hebert P.D. 2016. DNA barcodes from century-old type specimens using next-generation sequencing. Mol. Ecol. Resour. 16:487–497.

Puillandre N., Macpherson E., Lambourdière J., Cruaud C., Boisselier-Dubayle M.C., Samadi S. 2011. Barcoding type specimens helps to identify synonyms and an unnamed new species in *Eumunida* Smith, 1883 (Decapoda: Eumunididae). Invertebr. Syst. 25:322–333.

Rancilhac L., Bruy T., Scherz M.D., Pereira E.A., Preick M., Straube N., Lyra M.L., Ohler A., Streicher J.W., Andreone F., Crottini A., Hutter C.R., Randrianantoandro J.C., Rakotoarison A., Glaw F., Hofreiter M., Vences M. 2020. Target-enriched DNA sequencing from historical type material enables a partial revision of the Madagascar giant stream frogs (genus *Mantidactylus*). J. Nat. Hist. 54:87–118.

Rázuri-Gonzales E., Ngera M.F., Pauls S.U. 2022. A new species of *Silvatares* (Trichoptera, Pisuliidae) from the Democratic Republic of the Congo. ZooKeys. 371.

Richards J.L., Saunders G.W., Hughey J.R., Gabrielson P.W. 2021a. Reinstatement of Indian Ocean *Porolithon coarctatum* and *P. gardineri* based on sequencing type specimens, and *P. epiphyticum* sp. nov. (Corallinales, Rhodophyta), with comments on subfamilies Hydrolithoideae and Metagoniolithoideae. Bot. Mar. 64:363–377.

Richards J.L., Schmidt W.E., Fredericq S., Sauvage T., Peña V., Le Gall L., Mateo-Cid L.E., Mendoza-González A.C., Hughey J.R., Gabrielson P.W. 2021b. DNA sequencing of type material and newly collected specimens reveals two heterotypic synonyms for *Harveylithon munitum* (Metagoniolithoideae, Corallinales, Rhodophyta) and three new species. J. Phycol. 57:1234–1253.

Rogers S.O., Bendich A.J. 1985. Extraction of DNA from milligram amounts of fresh, herbarium and mummified plant tissues. Plant Mol. Biol. 5:69–76.

Rohland N., Hofreiter M. 2007. Ancient DNA extraction from bones and teeth. Nat. Protoc. 2:1756–1762.

Rohland N., Siedel H., Hofreiter M. 2004. Nondestructive DNA extraction method for mitochondrial DNA analyses of museum specimens. BioTechniques. 36:814–821.

Rohland N., Siedel H., Hofreiter M. 2010. A rapid column-based ancient DNA extraction method for increased sample throughput. Mol. Ecol. Resour. 10:677–683.

Ronikier A., Janik P., Haan M., Kuhnt A., Zankowicz M. 2022. Importance of type specimen study for understanding genus boundaries—taxonomic clarifications in *Lepidoderma* based on integrative taxonomy approach leading to resurrection of the old genus *Polyschismium*. Mycologia. 114:1008–1031.

Rougerie R., Haxaire J., Kitching I.J., Hebert P.D. 2012. DNA barcodes and morphology reveal a hybrid hawkmoth in Tahiti (Lepidoptera: Sphingidae). Invertebr. Syst. 26:445–450.

Russell D.W., Sambrook J. 2001. Molecular cloning: a laboratory manual. Cold Spring Harbor, NY: Cold Spring Harbor Laboratory.

Sambrook J., Fritsch E.F., Maniatis T., editors. 1989. Molecular cloning: a laboratory manual. Plainview, NY: Cold Spring Harbor Laboratory Press.

Scherz M.D., Rasolonjatovo S.M., Köhler J., Rancilhac L., Rakotoarison A., Raselimanana A.P., Ohler A., Preick M., Hofreiter M., Glaw F., Vences M. 2020. 'Barcode fishing' for archival DNA from historical type material overcomes taxonomic hurdles, enabling the description of a new frog species. Sci. Rep. 10:19109.

Schipper S.R., Shivak J.P., Hind K.R., Miller K.A., Hughey J.R., Gabrielson P.W., Martone P.T. 2023. Reinstatement of *Corallina chilensis* (Corallinaceae, Rhodophyta) based on DNA sequencing of the type material collected by Darwin. Phycologia. 62:203–216.

Schöneberg Y., Winter S., Arribas O., Di Nicola M.R., Master M., Owens J.B., Rovatsos M., Wüster W., Janke A., Fritz U. 2023. Genomics reveals broad hybridization in deeply divergent Palearctic grass and water snakes (*Natrix* spp.). Mol. Phylogenet. Evol. 184:107787.

Schweizer M., Etzbauer C., Shirihai H., Töpfer T., Kirwan G.M. 2020. A molecular analysis of the mysterious Vaurie's Nightjar *Caprimulgus centralasicus* yields fresh insight into its taxonomic status. J. Orn. 161:635–650.

Shumskaya M., Mironov K.S., Ballesteros J.A., Safonov I., Halling R.E. 2023. DNA isolation and genome sequence of the 134-year-old holotype specimen of *Boletus subvelutipes* Peck. Ecol. Evol. 13:10389.

Silva P.C., Malabarba M.C., Vari R., Malabarba L.R. 2019. Comparison and optimization for DNA extraction of archived fish specimens. MethodsX. 6:1433–1442.

Speidel W., Hausmann A., Mueller G.C., Kravchenko V., Mooser J., Witt T.J., Hebert P.D. 2015. Taxonomy 2.0: Sequencing of old type specimens supports the description of two new species of the *Lasiocampa decolorata* group from Morocco (Lepidoptera, Lasiocampidae. Zootaxa. 3999:401–412.

Stelbrink B., Kehlmaier C., Wilke T., Albrecht C. 2019. The near-complete mitogenome of the critically endangered *Pseudocleopatra dartevellei* (Caenogastropoda: Paludomidae) from the Congo River assembled from historical museum material. Mitochondrial DNA Part B. 4:3229–3231.

Straube N., Lyra M.L., Paijmans J.L., Preick M., Basler N., Penner J., Rödel M.-O., Westbury M.V., Haddad C.F.B., Barlow A., Hofreiter M. 2021a. Successful application of ancient DNA extraction and library construction protocols to museum wet collection specimens. Mol. Ecol. Resour. 21:2299–2315.

Straube N., Preick M., Naylor G.J.P., Hofreiter M. 2021b. Mitochondrial DNA sequencing of a wet-collection syntype demonstrates the importance of type material as genetic resource for lantern shark taxonomy (Chondrichthyes: Etmopteridae). R. Soc. Open Sci. 8:210474.

Strutzenberger P., Brehm G., Fiedler K. 2012. DNA Barcode Sequencing from Old Type Specimens as a Tool in Taxonomy: A Case Study in the Diverse Genus Eois (Lepidoptera: Geometridae). PLOS ONE. 7:e49710.

Stuart B.L., Fritz U. 2008. Historical DNA from museum type specimens clarifies diversity of Asian leaf turtles (*Cyclemys*). Biol. J. Linn. Soc. 94:131–141.

Sullivan J.P., Hopkins C.D., Pirro S., Peterson R., Chakona A., Mutizwa T.I., Dillman C.B. 2022. Mitogenome recovered from a 19th Century holotype by shotgun sequencing supplies a generic name for an orphaned clade of African weakly electric fishes (Osteoglossomorpha, Mormyridae). ZooKeys.:163.

Suzuki M., Segawa T., Mori H., Akiyoshi A., Ootsuki R., Kurihara A., Nozaki H. 2016. Next-Generation Sequencing of an 88-Year-Old Specimen of the Poorly Known Species *Liagora japonica* (Nemaliales, Rhodophyta) Supports the Recognition of *Otohimella* gen. Nov PLoS One. 11:0158944.

Taylor R.L., Bailey J.C., Freshwater D.W. 2017. Systematics of *Cladophora* spp. (Chlorophyta) from North Carolina, USA, based upon morphology and DNA sequence data with a description of *Cladophora subtilissima* sp. nov. J. Phycol. 53:541–556.

Twort V.G., Minet J., Wheat C.W., Wahlberg N. 2021. Museomics of a rare taxon: placing Whalleyanidae in the Lepidoptera Tree of Life. Syst. Entomol. 46:926–937.

Vaglia T., Haxaire J., Kitching I.J., Meusnier I., Rougerie R. 2008. Morphology and DNA barcoding reveal three cryptic species within the *Xylophanes neoptolemus* and loelia species-groups (Lepidoptera: Sphingidae). Zootaxa. 1923:18–36.

Vences M., Köhler J., Andreone F., Craul A.-K., Crottini A., du Preez L., Preick M., Rancilhac L., Rödel M.-O., Scherz M.D. 2021. Target-enriched DNA sequencing clarifies the identity of name-bearing types of the *Gephyromantis plicifer* complex and reveals a new species of mantellid frog from Madagascar. Spixiana. 44:175–202.

Vershinina A.O., Kapp J.D., Baryshnikov G.F., Shapiro B. 2020. The case of an arctic wild ass highlights the utility of ancient DNA for validating problematic identifications in museum collections. Mol. Ecol. Resour. 20:1182–1190.

Vieira C., Camacho O., Wynne M.J., Mattio L., Anderson R.J., Bolton J.J., Clerck O. 2016. Shedding new light on old algae: matching names and sequences in the brown algal genus *Lobophora* (Dictyotales, Phaeophyceae). Taxon. 65:689–707.

Wade R.M., Gabrielson P.W., Hind K.R., Shivak J., Hughey J.R., Ohtsu S., Baba M., Kogame K., Lindstrom S.C., Miller K.A., Schipper S.R., Martone P.T. 2023. Resolving some of the earliest names for *Corallina* species (Corallinales, Rhodophyta) in the North Pacific by sequencing type specimens and describing the cryptic *C. hakodatensis* sp. nov. and *C. parva* sp. nov. J. Phycol. 59:221–235.

Wilke T., Kehlmaier C., Stelbrink B., Albrecht C., Bouchet P. 2023. Historical DNA solves century-old mystery on sessility in freshwater gastropods. Mol. Phylogenet. Evol. 185:107813.
